# Characterization of the Ca^2+^-Gated and Voltage-Dependent K^+^-Channel Slo-1 of Nematodes and Its Interaction with Emodepside

**DOI:** 10.1371/journal.pntd.0003401

**Published:** 2014-12-18

**Authors:** Daniel Kulke, Georg von Samson-Himmelstjerna, Sandra M. Miltsch, Adrian J. Wolstenholme, Aaron R. Jex, Robin B. Gasser, Cristina Ballesteros, Timothy G. Geary, Jennifer Keiser, Simon Townson, Achim Harder, Jürgen Krücken

**Affiliations:** 1 Institute for Parasitology and Tropical Veterinary Medicine, Freie Universität Berlin, Berlin, Germany; 2 Global Drug Discovery, Animal Health, Parasiticides, Bayer HealthCare, Leverkusen, Germany; 3 Department of Infectious Diseases, College of Veterinary Medicine, The University of Georgia, Athens, Georgia, United States of America; 4 Faculty of Veterinary Science, The University of Melbourne, Parkville, Victoria, Australia; 5 Institute of Parasitology, McGill University, Sainte-Anne-de-Bellevue, Quebec, Canada; 6 Department of Medical Parasitology and Infection Biology, Swiss Tropical and Public Health Institute, Basel, Switzerland; 7 Tropical Parasitic Diseases Unit, Northwick Park Institute for Medical Research, Harrow, Middlesex, United Kingdom; 8 WE Biology, Heinrich-Heine-Universität Düsseldorf, Düsseldorf, Germany; Rush University Medical Center, United States of America

## Abstract

The cyclooctadepsipeptide emodepside and its parent compound PF1022A are broad-spectrum nematicidal drugs which are able to eliminate nematodes resistant to other anthelmintics. The mode of action of cyclooctadepsipeptides is only partially understood, but involves the latrophilin Lat-1 receptor and the voltage- and calcium-activated potassium channel Slo-1. Genetic evidence suggests that emodepside exerts its anthelmintic activity predominantly through Slo-1. Indeed, *slo-1* deficient *Caenorhabditis elegans* strains are completely emodepside resistant. However, direct effects of emodepside on Slo-1 have not been reported and these channels have only been characterized for *C. elegans* and related Strongylida. Molecular and bioinformatic analyses identified full-length Slo-1 cDNAs of *Ascaris suum*, *Parascaris equorum*, *Toxocara canis*, *Dirofilaria immitis*, *Brugia malayi*, *Onchocerca gutturosa* and *Strongyloides ratti*. Two paralogs were identified in the trichocephalids *Trichuris muris*, *Trichuris suis* and *Trichinella spiralis*. Several splice variants encoding truncated channels were identified in *Trichuris* spp. Slo-1 channels of trichocephalids form a monophyletic group, showing that duplication occurred after the divergence of Enoplea and Chromadorea. To explore the function of a representative protein, *C. elegans* Slo-1a was expressed in *Xenopus laevis* oocytes and studied in electrophysiological (voltage-clamp) experiments. Incubation of oocytes with 1-10 µM emodepside caused significantly increased currents over a wide range of step potentials in the absence of experimentally increased intracellular Ca^2+^, suggesting that emodepside directly opens *C. elegans* Slo-1a. Emodepside wash-out did not reverse the effect and the Slo-1 inhibitor verruculogen was only effective when applied before, but not after, emodepside. The identification of several splice variants and paralogs in some parasitic nematodes suggests that there are substantial differences in channel properties among species. Most importantly, this study showed for the first time that emodepside directly opens a Slo-1 channel, significantly improving the understanding of the mode of action of this drug class.

## Author Summary

Emodepside is an anthelmintic introduced into the market as an ingredient of different dewormers for cats and dogs, and is the only member of the cyclooctadepsipeptide class which has been commercialized. The voltage-gated and calcium-activated potassium channel Slo-1 has been implicated in the mode of action of emodepside, but evidence for direct emodepside-induced changes on the properties of Slo-1 channels has not been reported. Emodepside is active against a broad spectrum of parasitic nematodes, but the putative target Slo-1 has only been described for the model nematode *Caenorhabditis elegans,* and for relatively closely related strongylids. Here, Slo-1 channels of ascarids, filariae and trichocephalids are described. Unexpectedly, in the latter group two distinct Slo-1 channels are encoded in their genomes. The *C. elegans* Slo-1a channel was expressed in *Xenopus laevis* oocytes. After membrane depolarization without experimentally increasing intracellular Ca^2+^, no Slo-1 specific currents were observed. In contrast, very large Slo-1-specific currents were observed after preincubation of oocytes with emodepside, suggesting that the drug opens the *C. elegans* Slo-1a channel irreversibly. This is the first report demonstrating direct interaction of a cyclooctadepsipeptide with a Slo-1 channel, which substantially enhances our understanding of the mode of action of this drug class.

## Introduction

In Ecdysozoa, large conductance potassium channels (also BK or Maxi-K channels) are encoded by *slo-1* genes [1]. Due to their large conductance in the open state, typically exceeding 200 pS, Slo-1 channels are of major importance for repolarization of excitable cells. These channels are tetramers and, in almost every animal species, all subunits are encoded by a single gene – with teleost fish as the only known exception, encoding multiple *slo-1* paralogs resulting from whole genome duplications [2]. The opening of Slo-1 channels is controlled by the membrane potential and intracellular free Ca^2+^-concentrations [Ca^2+^]_i_. Depolarization of the membrane as well as very high transient local Ca^2+^ concentrations are required to open Slo-1 channels [3]. Most other ion channels produce subtypes with altered physiological properties through a combination of different subunits as recently reviewed for acetylcholine and glutamate receptors [4], [5]. In contrast, several different *slo-1* splice variants have been described, *e.g.* for *C. elegans*, *D. melanogaster*, mice and humans, and differential splicing is known to affect channel properties [6]–[9]. In addition, the use of different tissue-specific promoters has been described for *D. melanogaster* [10], [11]. Indeed, the number of different splice variants has dramatically increased during evolution, which is demonstrated by the fact that the human *slo-1* ortholog *kcnma1* encodes 13 alternative exons [12]. In fact, the number of splice variants in Mammalia and Diptera is so high that systematic analysis of the effects of the channel variants generated by alternative splicing on voltage- and Ca^2+^-sensitivity has only been performed for a few of them. The complexity of Slo-1 channel heterogeneity is further increased by the ability to form heterotetramers [13] containing different splice variants and the fact that channel responses to depolarization and Ca^2+^ are modulated by posttranslational modifications such as palmitoylation and phosphorylation at several distinct sites [14]–[16].

Splice variants in *C. elegans* are more clearly arranged, with four different splice sites giving rise to 12 well characterized splice variants [17] plus three additional variants annotated in WormBase (Slo-1a-Slo-1m, Slo-1y, Slo-1z). Expression levels of these variants in whole *C. elegans* as well as voltage- and Ca^2+^-sensitivity profiles of different homomeric tetramers have been reported recently [6], [17].

Nematode Slo-1 channels have received particular attention in recent years due to their central involvement in the mode of action of the broad-spectrum nematicidal cyclooctadepsipeptide emodepside [18]. *C. elegans* strains with *slo-1* loss-of-function mutations are completely resistant to emodepside [19]. Emodepside sensitivity can be rescued by re-introducing Slo-1 from either *C. elegans* or the parasitic nematodes *Ancylostoma caninum* and *Cooperia oncophora*, but only partially by the human ortholog KCNMA1 [19]–[21]. These results can be explained by either a direct and specific interaction of emodepside with nematode Slo-1 channels or a necessary function of Slo-1 in a signal transduction pathway downstream of an emodepside target. Ectopic expression of Slo-1a in the pharynx muscle, which does normally not express Slo-1 channels, in a *slo-1* deficient genetic background conferred emodepside sensitivity to pharyngeal pumping [21], suggesting that this channel is either the direct target of the drug or that an unidentified target is present in pharyngeal muscle cells that is activated upstream of Slo-1. Although Slo-1 channels are widely considered to be the most likely receptors for emodepside in nematodes [18], [22]–[24], direct interaction of the channel with emodepside or its activation by this drug have not been reported. This is in contrast to another putative emodepside target, the G protein-coupled receptor Lat-1, an ortholog of mammalian latrophilin receptors. Binding of the drug to its target and activation of Lat-1 by PF1022A and emodepside have been demonstrated [25]. However, *lat-1* loss-of-function mutations in *C. elegans* cause only partial emodepside resistance; the effects of emodepside on the pharynx but not on the body muscle were impaired [26]–[28].

Knowing the mode of action of new drugs has important advantages for the prediction of efficacy in new target species [29] and can exclude potential receptor-dependent deleterious side effects in host species [30]. Interpretation of structure-activity relationships are also facilitated if the receptor is known. Despite their relatively uniform body shape, nematodes are genetically and physiologically extremely diverse [31], [32]. The cyclooctadepsipeptides have a very broad anthelmintic spectrum against parasitic nematodes [18] representing all major clades [33], including strongylids (clade V), *Strongyloides* (clade IV), Ascaridoidea and Filarioidea (clade III) as well as the trichocephalids *Trichuris* and *Trichinella* (clade I) [18]. This broad spectrum nematicidal activity is in marked contrast to other anthelmintics that have been developed in the last decade including tribendimidine (poor efficacy against *Strongyloides stercoralis* and *Trichuris trichiura* [34]), monepantel (only limited efficacy against human hookworms and poor or no efficacy against parasites not belonging to clade V [35]) as well as derquantel (efficacy >95% against L4 and adults of *Trichostrongylus* and *Nematodirus* spp. as well as against adult *H. contortus* but suboptimal efficacies against L4 and adults of *Teladorsagia circumcincta*, L4 of *H. contortus*, and some large intestinal nematodes [36]).

For *S. ratti* lack of monepantel efficacy is presumably due to the fact that no member the DEG-3/DES-2 subfamily of acetylcholine receptors, which are the known targets of this drug, are encoded in its genome [29]. Therefore, differences in susceptibility to anthelmintics among nematodes can in part be explained by presence of drug targets encoded in their genomes.

*slo-1* genes and gene products have thus far only been investigated in clade V of the phylum Nematoda [33]. Since parasitic nematodes of vertebrates are found in four of five clades [33] and Slo-1 is apparently a validated drug target, the present study aimed to analyze the diversity of Slo-1 channels within the phylum Nematoda. In addition, effects of emodepside on *C. elegans* Slo-1a channel opening were determined.

## Materials and Methods

### Ethical statement

All animal experiments were approved by the local administrations in charge and were in accordance with local laws regarding animal welfare (Animal Welfare Act in the United States and the “Tierschutzgesetze” in Germany and in Switzerland as well as in accordance with the European Union directive 2010/63/EU). Adult *T. muris* were obtained during controlled drug trials performed for other studies at Bayer HealthCare AG, Global Drug Discovery Animal Health in Monheim, Germany (approved by Landesamt für Natur-, Umwelt- und Verbraucherschutz Nordrhein-Westfalen (LANUV) in Recklinghausen (Germany) under No. 200/V14) [37] and at the Swiss Tropical and Public Health Institute in Basel, Switzerland (approved by the local veterinary office Basel-Stadt (Switzerland) based on Swiss cantonal and national regulations under permission no. 2070) [38]. Adult *Parascaris equorum* were collected in a controlled drug trial study conducted by von Samson-Himmelstjerna *et al.* that was approved by the Landesamt für Verbraucherschutz und Lebensmittelsicherheit (LAVES) in Hannover (Germany) under the reference number 33.9-42502-05-07A499. *Ascaris suum* were obtained from a German slaughterhouse. *Dirofilaria immitis* macrofilariae were collected during routine necropsy of a naturally infected, moribund stray dog which was euthanized due to medical reasons (Athens, GA, USA). No information regarding owners or the history of the dog is available. Macrofilariae of *Onchocerca gutturosa* were dissected from the nuchal ligament connective tissues, obtained from cattle post-slaughter in Banjul, Gambia, (facilitated by the International Trypanotolerance Centre, Gambia). Consent of the owners of the carcasses was obtained. *Brugia malayi* microfilariae were provided by the NIAID/NIH Filariasis Research Reagent Resource center (FR3). *Toxocara canis* RNA was obtained from a previously published study [39].

### RNA isolation and cDNA synthesis

Nematodes were homogenized on ice in Trizol (Invitrogen) or TriFast (Peqlab) reagent using a TissueRuptor and transparent disposable probes (Qiagen). RNA was isolated according to the manufacturer's instructions, except that the volume of Trizol was increased to 5 ml per 100 mg wet tissue weight. Volumes of all subsequently used reagents were adjusted accordingly. RNAs were precipitated twice in the presence of glycogen (Thermo Fisher Scientific), dissolved in water and stored at -80°C until use.

For cDNA synthesis, 1 µg total RNA was mixed with 100 pmol of either random hexamer primers (for PCR with degenerate primers) or oligo dT primers (for full-length PCRs; both primers from Thermo Fisher Scientific), incubated at 65°C for 5 min and then chilled on ice. Reverse transcription was performed in 20 µl using 200 U Maxima Reverse Transcriptase (Thermo Fisher Scientific), 1 mM dNTPs and 20 U RiboLock RNase inhibitor (Thermo Fisher Scientific) by incubation at 42 °C for 30 min and 60 °C for 30 min. Finally, the enzyme was inactivated at 70 °C for 5 min. Alternatively, 200 U RevertAidM-MuLV reverse transcriptase (Thermo Fisher Scientific) were used with 20 U RiboLock RNase inhibitor, 0.5 mM dNTPs and 25 µM oligo-dT primers.

### Amplification of full-length cDNAs

Partial genomic data from *T. muris* and *T. canis* and a deduced full-length sequence of *S. ratti* (for phylogenetic analysis only) were provided by the Parasite Genomics group at the Wellcome Trust Sanger Institute and can be obtained from http://www.sanger.ac.uk/research/projects/parasitegenomics/.

Based on comparisons among available slo-1 cDNA sequences of *H. contortus*, *C. oncophora* and *C. elegans,* degenerate primers were designed and used to amplify and sequence small slo-1 fragments of *P. equorum* and *A. suum*. A 126 bp long cDNA *T. canis slo-1* sequence published on nematode.net [40] was identified by BLAST. Specific primers for nested 5'- and 3'-RACE PCR were designed using these sequence data (S1 Table). PCRs contained 0.3 mM dNTPs, 0.4 µM of the gene-specific and the universal primer, 1 µl cDNA and 0.5 µl Advantage 2 Polymerase Mix (Clontech) in 25 µl 1×Advantage buffer. For 5'-RACE multiple nested RACE PCRs were performed to proceed stepwise to the 5'-end of the cDNA. Full-length sequences were amplified using two gene-specific primers and the same PCR protocol. PCR fragments were gel purified, cloned into pCR4 TOPO (Invitrogen) and sent to GATC Biotech for sequencing. Primers for all full-length amplifications are provided in S1 Table.

For other nematodes, primers derived from partial *slo-1* sequences identified in the *T. muris*, *D. immitis*, *Onchocerca volvulus* and *B. malayi* genome projects were used. Using these sequences, primers for 5' and 3' RACE PCR were designed and RACE PCRs were carried out with the 5'/3' RACE Kit, second generation (Roche Diagnostics) as detailed previously [39]. PCRs were performed in 25 µl 1×Phusion II buffer containing 1 U Phusion II DNA polymerase (Thermo Fisher), 0.3 mM dNTPs, 0.3 µM of each primer, 1×Q solution (Qiagen) and 1 µl cDNA synthesized with RevertAidM-MuLV reverse transcriptase. Thermocycling was conducted in a Biorad C1000 or S1000 cycler with initial denaturation at 98°C for 1 min followed by 35 cycles with denaturation at 98°C for 10 s, annealing at a primer pair specific temperature for 30 s and elongation at 72°C for 20 s to 2 min. For full-length PCRs, primers flanking the open reading frame were chosen. Full-length primers for *B. malayi*, *D. immitis* and *O. gutturosa* were obtained from genome data [41]. Further details about primer sequences are available in S1 Table. PCR products were gel-purified and cloned into the pCR4 TOPO blunt vector (Invitrogen) and sequenced by GATC Biotech.

### Identification of protein motifs in Slo-1 channels

Molecular weight and putative isoelectric points were calculated with Clone Manager 9 (Scientific and Educational Software). Localization of transmembrane regions was predicted using TMPred software [42]. Conserved domains and Prosite motifs were identified using CD-BLAST [43], [44] and InterProScan [45], [46]. Prediction of phosphorylation sites was performed using NetPhosK 1.0 [47].

### Phylogenetic analysis

Slo-1 sequences from the present study were aligned with homologs available in GenBank or Wormbase, and homologs from *Meloidogyne incognita* [48] and *S. ratti* (obtained from the Sanger genome project). Deduced protein sequences were aligned using ClustalX2 [49]. Alignments were analyzed with Prottest 3.0.1 [50] to identify the most appropriate amino acid substitution model. PhyML 3.0.1 [51], [52] was then used to determine tree topology and branch support as described [53]. In brief, the JTT model for amino acid substitution [54] with 16 Γ distributed substitution rate categories was used. PhyML was set to estimate Γ shape parameter and proportion of invariable sites while amino acid frequencies were set to be based on the substitution model. Nearest neighbor interchange (NNI) and subtree pruning and regraftment (SPR) moves were allowed to optimize the tree topology. Both Bayesian transformation and Shimodaira-Hasegawa-like modifications of the approximate likelihood test were used to calculate branch support. Tree optimization started with one neighbor-joining and 5 random trees and the tree with the highest likelihood was finally chosen and visualized in MEGA5 [55].

For analysis of the conserved alternative exon, ProtTest 3.0 identified the same model as for the full-length sequence and PhyML was executed using identical parameters as for the full-length sequence.

### Experimental detection of differential splice products

The primers flanking the putative splice site (S1 Table) were used in a two-step RT-PCR as described above. PCR products were analyzed on 2.0% agarose gels and with the DNA 1000 kit on the Bioanalyzer 2100 (Agilent). Gel-purified DNA fragments were cloned and sequenced. Quantification of fragments was performed using the Bioanalyzer Expert software.

### *Trichuris suis* transcriptome data

For RNA-seq experiments, total RNAs were extracted from larvae at day 10 [approximately 50,000 first and second stage larvae (L1 and L2) from 5 experimentally infected pigs], day 18 [15,000 third stage larvae (L3) from 4 pigs] and day 28 p.i. [3,000 fourth stage larvae (L4) from 2 pigs], from whole adult male (n = 10), adult female (n = 10) or from multiple stichosomes (mixed sex; n = 10), posterior portions of adult females (n = 10), including the eggs, and of adult males (n = 10) using the TriPure reagent (Roche). Yield and quality were verified using the 2100 Bioanalyzer (Agilent). For library production, purification of polyadenylated (polyA^+^) RNA from 10 µg total RNA from each sample was carried out using Sera-mag oligo(dT) beads (Thermo Scientific). Then RNAs were fragmented to a size of 300–500 bp, reverse-transcribed using random hexamer primers, end-repaired and adapter-ligated according to the manufacturer's protocol (Illumina). Ligation products of approximately 400 bp were eluted from agarose gels, PCR-amplified (15 cycles) as recommended and purified on MinElute columns (Qiagen). Finally, libraries were subjected to paired-end RNA-seq using HiSeq 2000 (Illumina) and assessed for quality and adapter sequences. After sequencing, raw reads were trimmed of Illumina adapters, filtered for length (≥ 40 nt) and low-quality data (reads containing 4 or more consecutive bases showing a PHRED quality below 20). Transcripts for each stage and/or body portion as well as the Illumina RNA-seq data from a mixed-sex adult *T. suis* transcriptome generated previously [56] were reconstructed and quantified by Jex et al. [57] using the Tuxedo suite [58] and a draft assembly of the *T. suis* genome [57]. Differential transcription was assessed using NOISeq [59], with 20% of the evaluated reads for each library used in 5 iterations to simulate technical replicates (note: all RNA-seq libraries were constructed from multiple parasites, i.e., 10 adults or 50,000 larvae retrieved from multiple host individuals to compensate for inter-individual biological variation).

### Preparation of slo-1 cRNAs

Full-length cDNAs encoding *C. elegans* Slo-1a and *T. muris* Slo-1.1a in pCR4TOPO (Invitrogen) were used. Plasmid DNA was linearized in 100 µl buffer containing 8 µg plasmid DNA and 100 U XbaI (for *C.elegans* SLO-1a) or 100 U BcuI (for *T. muris* SLO-1.1a) at 37°C for 2 h. Linearized plasmid DNA was purified via the GeneJet PCR Purification kit (Thermo Fisher Scientific) and eluted with 50 µl UltraPure DNase/RNase-free distilled water. Linearization of the plasmid DNA was monitored by agarose gel electrophoresis. DNA concentration was determined using the DNA 12000 kit and the 2100 Bioanalyzer (Agilent Technologies) according to the manufacturer's instructions. For cRNA synthesis from 1 µg linearized plasmid DNA, the mMessage mMachine T7 transcription kit (Ambion) was used according to manufacturer's instructions. To determine the integrity and concentration of the transcribed cRNA, the RNA 6000 Nano kit and the 2100 Bioanalyzer (Agilent Technologies) were used according to the manual.

### Microinjection of *Xenopus* oocytes

Defolliculated *Xenopus laevis* oocytes were obtained from EcoCyte Bioscience (Castrop-Rauxel, Germany). After delivery, single oocytes were transferred into individual cavities of a 48 well plate containing Barth's solution (88 mM NaCl, 2 mM KCl, 1 mM MgCl_2_, 15 mM Tris HCl, 0.5 mM CaCl_2_, pH 7.4) and incubated for at least 2 h at 19°C before use. 75 nl of either 200 ng/µL *C. elegans* SLO-1a cRNA or *T. muris* SLO-1.1a were microinjected into each oocyte using the Roboinject (Multi Channel Systems MCS GmbH). Oocytes injected with 75 nl water served as negative control. Position of impalement was set to 350 µm, whereas position of injection was set to 400 µm. Oocytes were incubated at 19°C for 3-4 days post microinjection. Barth's solution was replaced daily.

### Voltage clamp experiments with *Xenopus* oocytes

Experiments were carried out using the Roboocyte (Multi Channel Systems MCS GmbH). In the Roboocyte, oocytes were perfused with normal frog Ringer solution (NFR: 90 mM NaCl, 2 mM KCl, 2 mM CaCl_2_, 1 mM MgCl_2_, 5 mM HEPES, pH 7.4) for 1 min before electrodes were inserted and membrane potential was clamped to −70 mV. Experiments were carried out as summarized in S1 Fig.. In general, initial current-voltage curves (IVCs) were recorded after another perfusion for 1 min. Currents were recorded at a frequency of 1000 Hz during clamping the membrane potential between −120 and +60 mV for 500 ms using voltage steps of 20 mV (S1B Fig.) and mean currents for each voltage step were calculated from these 500 individual values. Between individual steps, the membrane potential was clamped to −70 mV for 3 s. After initial recordings of IVCs, a perfusion with NFR was performed for 2 min before repeating the recording to ensure that responses were stable over time (S1A(I) Fig.). Oocytes showing currents exceeding 250 nA at +60 mV were excluded from further experiments. To determine the effects of drugs, oocytes were first incubated with the vehicle (0.1% DMSO, 0.003% Pluronic F-68 (Sigma Aldrich) for 2 min. Vehicle, emodepside and blockers were always added manually into the well. IVCs were recorded and oocytes were again perfused for 1 min before incubation with emodepside (1 or 10 µM) and another recording (S1A(II) Fig.). Since the actual concentration of emodepside at its target site is completely unknown, 10 µM emodepside, which is the highest concentration soluble in water with 0.1% DMSO and 0.003% Pluronic F-68, was compared with 1 µM emodepside to identify any potential concentration dependent effects.

To test whether potential emodepside effects were reversible, oocytes were perfused for 5 to 10 min with NFR after an IVC recording in the presence of emodepside (S1A (III), (IV) Fig.). Effects of the blocker verruculogen (1 µM) were determined both before and after addition of emodepside (1 µM) to the oocytes (S1A (V), (VI) Fig.). Currents were recorded using Roboocyte ClampAmp software 2.2.0.15.

### Statistical analysis of electrophysiological data

Mean currents during each voltage step were calculated for individual oocytes in Roboocyte ClampAmp. To obtain IVCs, mean currents ± SEM for all replicate oocytes were plotted against the voltage used to clamp the membrane potential in GraphPad Prism 6.00. Mean currents between different groups of oocytes were compared separately for every step potential applied using the multiple t-test function in GraphPad Prism . P values were corrected for multiple comparisons using the Holm-Sidak method. Certain parts of the IVC were analyzed for different slopes and for linearity using the linear regression analysis in GraphPad Prism followed by the Wald-Wolfowitz runs test implemented in this analysis.

## Results

### Comparison of slo-1 cDNAs and encoded proteins

S2 Table summarizes the physico-chemical properties of predicted nematode Slo-1 subunits compared to homologous proteins from other nematodes and some orthologs from other species used as outgroup in further phylogenetic analysis. Only a single full-length cDNA sequence was identified from clade III nematodes (*B. malayi*, *O. gutturosa*, *P. equorum*, *A. suum* and *T. canis*), although alternative exons were identified in partial PCR products. These sequences were not included in the initial analysis of differential splicing since no information regarding possible combinations of different exons is currently available for them. For *B. malayi*, six different isoforms (*Bma*Slo-1c-h) are annotated in Wormbase and isoform *Bma*Slo-1f was identical to the one identified in the present study (S2 Fig.). For *D. immitis* two variants were identified differing only in a short region encoding amino acids 678 - 692 in *Dim*Slo-1a, which is missing in *Dim*Slo-1b (S2 Fig.).

In the clade I nematode *T. muris*, two partial genomic sequences encoding Slo-1 homologs were identified in contigs NODE_15952_length_5825_cov_10.724463 and NODE_133417_length_23144_cov_11.427541. Full-length cDNAs of both paralogs were amplified and cloned. Comparison with *Trichinella spiralis* sequences in GenBank revealed that this clade I nematode also encodes two distinct Slo-1 homologs in its genome. The *T. spiralis* Slo-1.1 protein sequence can be found as two partial entries which cover the whole length under the GenBank accession numbers XP_003370273.1 and XP_003370274. The *T. spiralis* Slo-1.2 database entries XP_003370270.1, XP_003370271.1 and XP_003370272.1 correspond to the *T. muris* Slo-1.2. In both trichocephalids, the two paralogs are juxtaposed in the genome in a tail-to-tail orientation.

**Figure 1:**
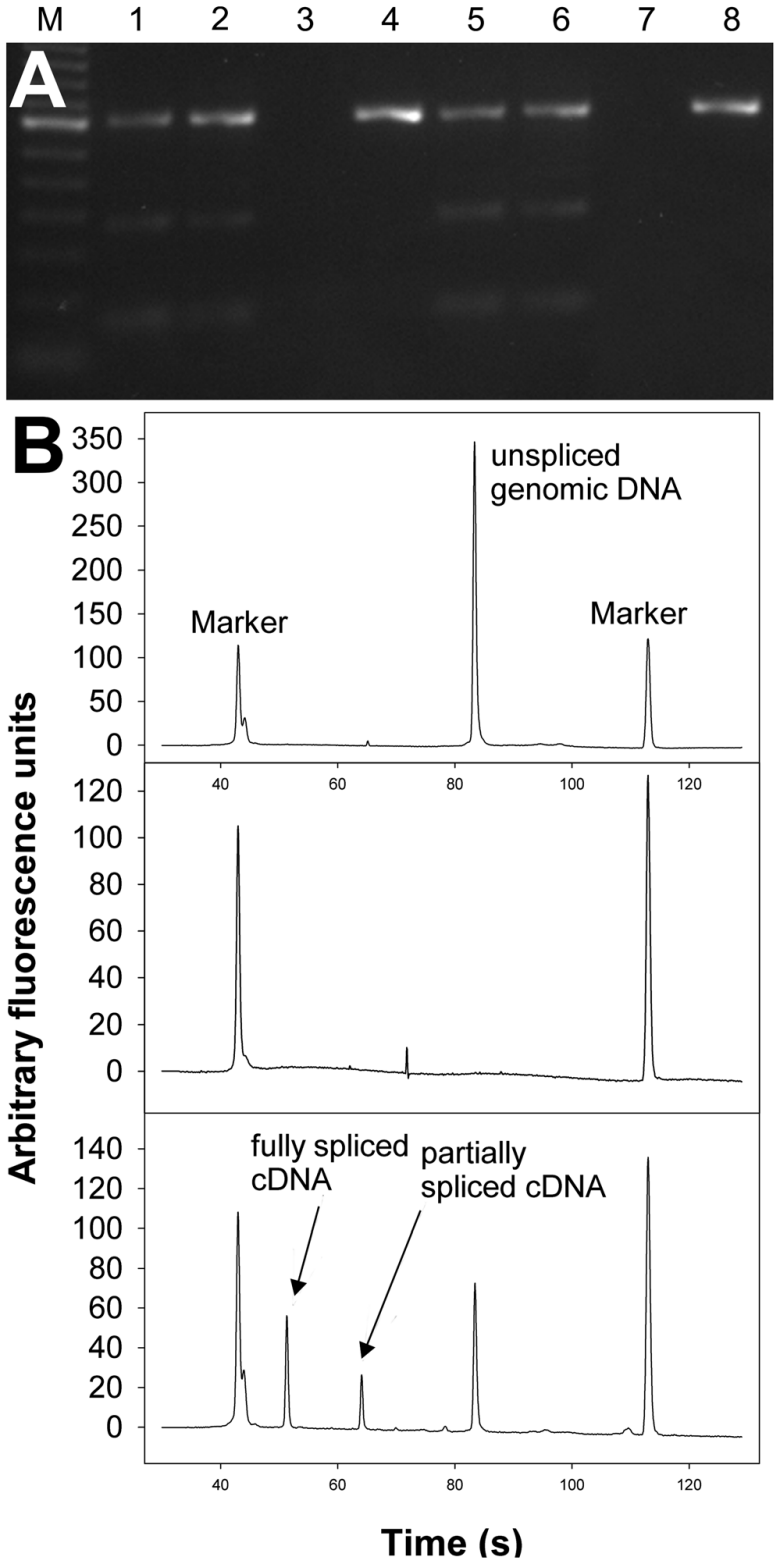
Estimation of splice variant abundances encoding truncated or full-length versions of *Trichuris muris* Slo-1.1. **A**) Primers flanking the introns retained in the cDNAs encoding the truncated proteins (*Tmu*Slo-1.1c and *Tmu*Slo-1.1d) were used in RT-PCR (lanes 1, 2, 5, and 6) and genomic PCR (lanes 4 and 8). Template was either derived from the Monheim (lanes 1–4) or the Edinburgh Zoo (lanes 5–8) isolate. Lanes 3 and 7 show no reverse transcription controls. M, 100 bp ladder. **B**) Representative samples separated on the Bioanalyzer. The upper panel shows a genomic PCR product, the middle panel the control without reverse transcription and the bottom panel the RT-PCR.

**Figure 2:**
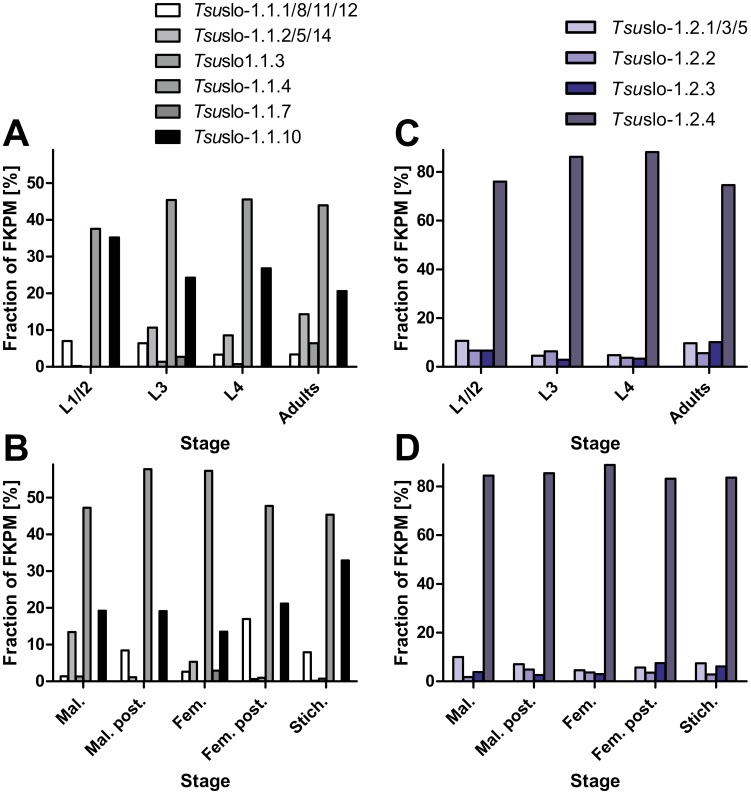
Frequencies of splice variants in terms of fragments per kilobase of exons per million fragments mapped (FKPM). Libraries were compared according to developmental stage (**A**) and (**C**) as well as different sexes or tissues (**B** and **D**). Splice variants were grouped according to the encoded protein, i.e. frequencies of splice variants encoding the same protein and differing in sequence downstream of the stop codon were added together. **A**) and **B**) show expression patterns of *Tsu*slo-1.1 and **C**) and **D**) indicate patterns for *Tsu*slo-1.2 splice variants. L1, L2, L3, L4, first, second, third, fourth stage larvae; Mal., males, Mal. post., posterior part of males; Fem., Females; Fem. post., posterior part of females; Stich., stichosome.

Two splice variants encoding full-length *T. muris* Slo-1.1 channels (Slo-1.1a and Slo-1.1b) were identified. In addition, two splice variants encoding severely truncated proteins were cloned; truncation is caused by retention of a partial or entire intron in the mature RNA leading to premature stop codons (*Tmu*Slo1.1c and *Tmu*Slo-1.1d in S2 Fig., respectively). Using primers flanking these introns, a semiquantitative estimate of the frequency of the splice variants was possible in two independent *T. muris* isolates. PCR products were separated on agarose gels (Fig. 1A) and the Bioanalyzer (Fig. 1B). The latter was used to quantify the amount of DNA in the peaks (Table 1). In contrast to the initial impression from agarose gels, where the large PCR products encoding the truncated versions produced the brightest bands, the Bioanalyzer clearly shows that the smallest fragment encoding full-length channels represents the majority of amplicons in terms of molecules (77 and 84%). To reveal if these truncated channel versions are present in related species, data from a *T. suis* genome/transcriptome project were analyzed for the presence of different slo-1 splice products. A multi sequence alignment of the encoded protein sequences is given in fasta format in S3 Fig.. Truncated versions due to retained introns were observed in both *Tsu*Slo-1.1 and *Tsu*Slo-1.2 in the same region of the channel (i.e., the transmembrane helix S4 and the voltage sensor comprised of S5 and S6), but the introns involved in these events were not at exactly the same position. Remarkably, alternative exons were found immediately before the retained introns, with one splice variant only present among cDNAs encoding full-length channels (*Tsu*Slo-1.1.4/7) and the alternative variant only in the truncated versions (*Tsu*Slo-1.1.2/5/10 and 14). In addition, splice variants encoding even shorter channels than those found in *T. muris* were identified (*Tsu*Slo-1.1.1/3/8/12). Quantitative analysis of transcriptome data revealed for *Tsu*slo.1.1 that the most abundantly detected splice variant corresponds to the full-length splice variant *Tsu*slo-1.1.4 in terms of fragments per kilobase of exons per million fragments mapped (FKPM), whereas the other full-length variant *Tsu*slo1.1-7 was found at much lower abundance (Fig. 2). The second most abundantly detected splice variant (*Tsu*slo1.1.10) encodes a severely truncated protein of similar length as the truncated *T. muris* variants detected by RT-PCR (S2 Fig.).

**Table 1:** Relative abundance of cDNA amplicons encoding full-length or truncated *Trichuris muris* Slo-1 channels.

	*T. muris* isolate			
	Monheim		Edinburgh Zoo	
-RT control	no amplicon		no amplicon	
Genomic PCR	353 bp	100%±0%	353 bp	100%±0%
RT-PCR	67 bp	77.6±0.6%	67 bp	83.8%±0.4%
	180 bp	7.8%±0.4%	180 bp	4.5%±1.7%
	353 bp	12.7%±0.6%	353 bp	11.6±2.0%

Remarkably, there are very obvious differences in frequency of splice variants.For example, the frequency of *Tsu*slo-1.1.10 ranged between 20% in adults and 35% in L1, with L3 and L4 showing intermediate frequencies (Fig. 2A). Additional stage specific splicing is exemplified by the channels encoded by *Tsu*slo-1.1.2/5/14 and *Tsu*slo-1.1.3: While the former encodes a channel very similar to Tsuslo-1.1.10, but differing in its NH_2_ terminus, the latter variant is extremely truncated (S2 Fig.). Notably, the abundance of *Tsu*slo-1.1.10 is relatively high among slo-1.1 transcripts during larval development, peaking in the L1/L2 stage (Fig. 2A). In contrast, transcription of this highly truncated slo-1.1 splice variant was relatively low (statistically significant at a false discovery rate of 0.1) in most adult libraries, with the major exception being the stichosome, in which it displayed its highest abundance. Other minor slo-1.1 isoforms, including some slo-1.1 variants, showed evidence of differential transcription during development and in adulthood among genders/tissues. However, because single library replicates were used for each tissue and stage of *T. suis*, statistical assessment of the differential transcription of these minor isoforms is not advisable. We consider these differences both preliminary and qualitative at this stage. In contrast to *Tsu*slo-1.1, little variation in transcription of *Tsu*slo-1.2 channel variants was observed among developmental stages or adult tissues/genders, with *Tsu*slo-1.2.6 (the full length transcript) being the major isoform (Fig. 2C and D).

Comparison of splice variants in parasitic nematodes with the 15 splice variants described for *C. elegans* reveals five main regions where differential splicing occurs (S2 Fig.). The first site is an alternative exon (i.e., present or not), the absence of which results in the use of a downstream ATG start codon only in *T. suis* Slo-1.1 and 1.2. Insufficient data are available to determine whether this splicing also occurs in *T. muris*. The first alternative exon identified in *C. elegans* (region 2 in S2 Fig.; also a ‘present or not’ type) was not found in any of the parasitic nematode slo-1 cDNAs. Splicing region 3 refers to a larger region where differential splicing occurs in all four *Trichuris* genes in different positions. Many but not all of the encoded proteins are severely truncated. The fourth splicing site (alternative exons) has been conserved throughout the evolution of Slo-1 channels in nematodes and two versions are present in *T. muris* and *T. suis* Slo-1.1. In all seven sequences cloned from ascarids and filariae, the corresponding region shows higher similarity to *C. elegans* Slo-1a. However, alternative sequences were identified in the genomes of *A. suum*, *O. volvulus* and *B. malayi* (*Bma*Slo-1h in S2 Fig. and S2 Table). Alternative splicing occurs in exactly the same position in arthropods and vertebrates. This alternative exon immediately follows two highly conserved phosphorylation sites. Remarkably, two of the predicted *B. malayi* splice variants contain a partial duplication (approximately two thirds of the whole exon *Bma*Slo-1c/d). Splicing site four in *C. elegans* (position 5 in S2 Fig.; ‘present or not’ type with two different 5' splice sites) has no equivalent in any of the *Trichuris* sequences, but there is alternative splicing in the same area in *B. malayi* and *D. immitis*. The sixth splice region is found in *C. elegans* but is apparently not present in clade III and clade I parasitic nematodes.

### Phylogenetic analysis of Slo-1 channels

**Figure 3:**
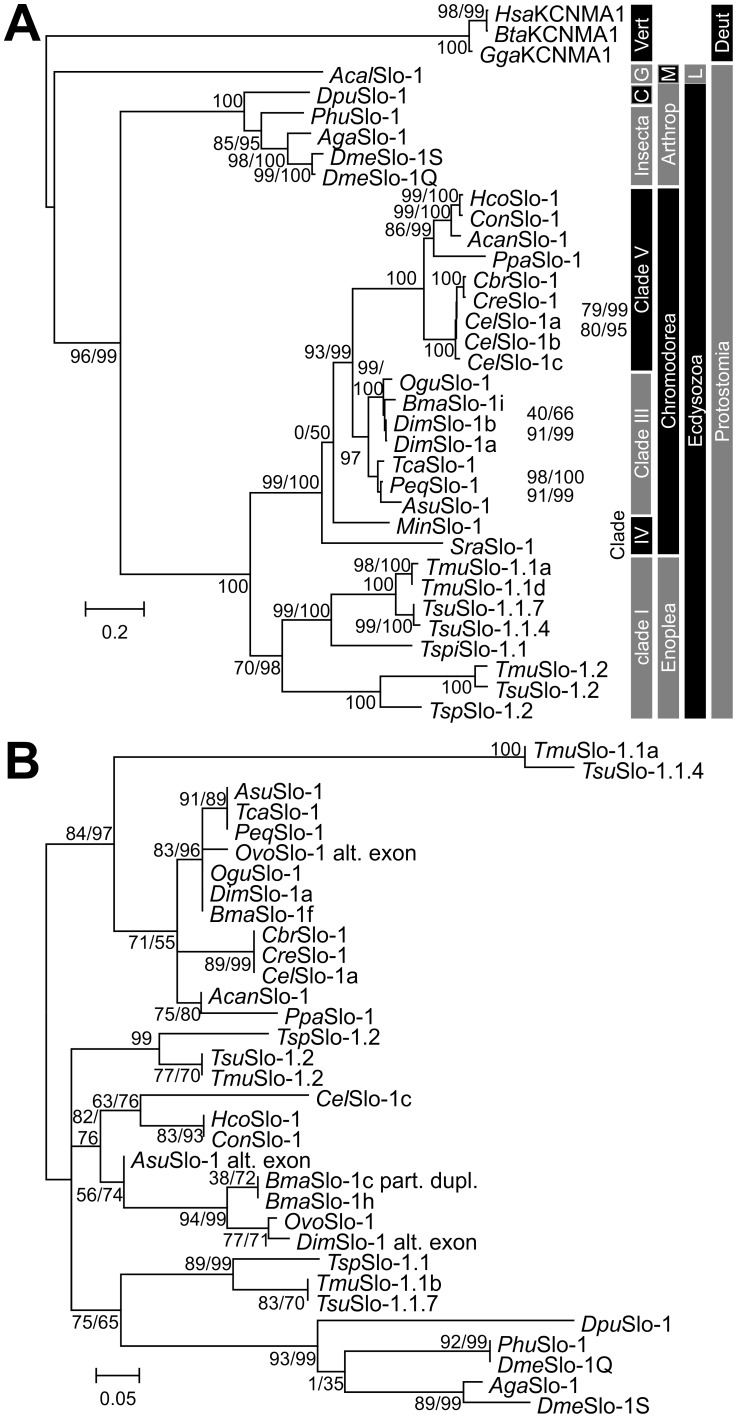
Phylogenetic analysis of nematode Slo-1 channels. **A**) Phylogram obtained by maximum likelihood analysis from full-length Slo-1 channels. Slo-1 protein sequences from the nematode species *Caenorhabditis elegans* (*Cel*), *Caenorhabditis briggsae* (*Cbr*), *Caenorhabditis remanei* (*Cre*), *Pristionchus pacificus* (*Pca*), *Haemonchus contortus* (*Hco*), *Cooperia oncophora* (*Con*), *Ancylostoma caninum* (*Acan*), *Onchocerca gutturosa* (*Ogu*), *Brugia malayi* (*Bma*), *Dirofilaria immitis* (*Dim*), *Toxocara canis* (*Tca*), *Parascaris equorum* (*Peq*), *Ascaris suum* (*Asu*), *Meloidogyne incognita* (*Min*), *Strongyloides ratti* (*Sra*), *Trichuris muris* (*Tmu*) and *Trichuris suis* (*Tsu*) were aligned together with orthologs from *Drosophila melanogaster* (*Dme*), *Anopheles gambiae* (*Aga*), *Pediculus humanus corporis* (*Phu*), *Daphnia pulex (Dpu)*, *Aplysia callifornica* (*Acal*), *Gallus gallus* (*Gal*), *Bos taurus* (*Bta*) and *Homo sapiens* (*Hsa*), which were used as outgroup, using ClustalX2. For *B. malayi* only the experimentally identified splice variant Slo-1f and for *C. elegans* only the variants Slo-1a-c were included. The JTT model of amino acid substitutions was used and PhyML was set to optimize the number of invariable sites while amino acid frequencies were based on the model. The number of Γ distributed substitution rate categories was set to 16 and PhyML optimized the Γ shape parameter. Support for individual nodes was calculated using the Shimodaira-Hasegawa modification and a Bayesian transformation of the approximate likelihood ratio test and results are shown close to the nodes before and after the slash, respectively. For those cases where support values were not shown next to the node they are shown on the right and refer to the most terminal node on the same vertical position. The scale bar represents 0.2 substitutions per site. C, Crustacea; G, Gastropoda; Vert, Vertebrata; Arthropod, Arthropoda, M, Mollusca; L, Lophotrophora; Deut, Deuerostomia. **B**) Phylogenetic tree calculated on an alignment of the conserved alternative exons from all Ecdysozoa included in the tree in A). In addition, four alternative exons identified in *Bma*Slo-1h and in the genome sequences of *Onchocerca volvulus* (*Ovo*Slo-1 and *Ovo*Slo-1 alt. exon) and *A. suum* (*Asu*Slo-1 alt. exon) were included. Parameters were identical to those used to calculate the tree from full-length sequences.

A maximum-likelihood approach was used to calculate a phylogenetic tree from the SLO-1 amino acid sequences (Fig. 3A). As outgroup, three sequences from vertebrates, one from a mollusc and five from arthropods were included in the analysis. Slo-1 channels of nematodes and arthropods formed a monophyletic group in accordance with their phylogenetic position within the Ecdysozoa. In general, most of the tree topology is consistent with current views on nematode evolution. However, the position of the Slo-1 proteins from the clade IV nematodes *M. incognita* and *S. ratti* is basal to a group containing all clade III and clade V sequences, but the support values for this position are rather low in both versions of the approximate likelihood test (Fig. 3A). The additional paralog Slo-1.2 present in clade I parasitic nematodes is placed as a sister operational taxonomic unit (OTU) to the Slo-1.1 paralog in this clade. This suggests that duplication of the *slo-1* gene occurred after the trichocephalids diverged from the other groups and is not an ancestral feature of nematode genomes.

To prove that differential splice variants were conserved throughout nematode evolution at the *C. elegans* alternative splice site four, only the amino acid sequences of this exon were aligned and subjected to phylogenetic analysis. For this purpose, alternative exons identified in the genomes of *A. suum* and *O. volvulus* were included. The partial duplication of this exon in *Bma*Slo-1c/d was also included as a separate OTU [*Bma*Slo-1c (part. dupl.) in S2 Fig]. Results are depicted in Fig. 3B. Due to the small size of the sequence, statistical support for individual nodes is lower than for full-length sequences. However, there is clearly a nematode-specific group with high similarity to the sequence in *C. elegans* Slo-1a, although the exon sequences in the trichocephalid species strongly diverge from those in clade III and V nematodes. The second group contains exon sequences from both nematodes and arthropods, including both alternative exons encoded in the *D. melanogaster* genome. Diversity within this group is much higher and deep divergence patterns could not be resolved. Therefore, the group consists of three major lineages, i.e. (i) *Trichuris* Slo-1.1b-like and arthropod sequences which might therefore be considered to be “ancestral”, (ii) the exon encoded in Slo-1.2 genes, (iii) the sequences derived from clade III and V nematodes.

### Voltage clamp experiments

**Figure 4:**
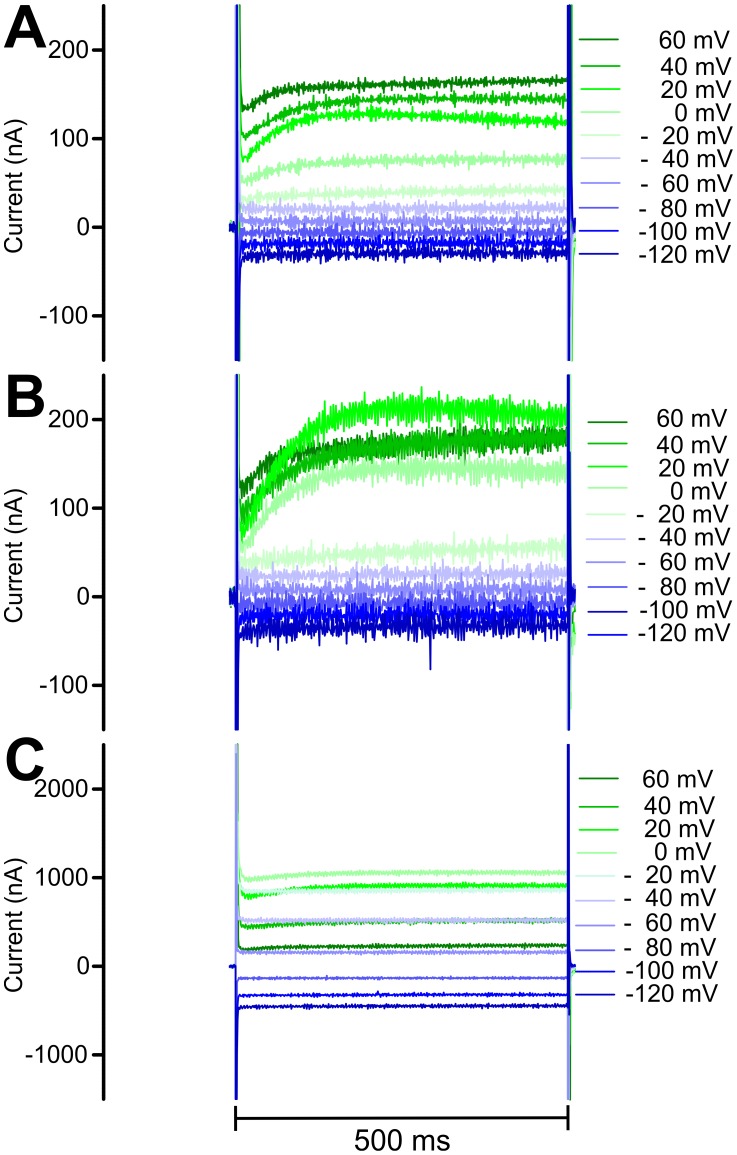
Currents observed in voltage-clamp experiments. Representative currents determined in oocytes injected with water (**A**), *Cel*slo-1a cRNA in the absence of emodepside (**B**) and *Cel*slo-1a cRNA in the presence of 10 µM emodepside (**C**).

**Figure 5:**
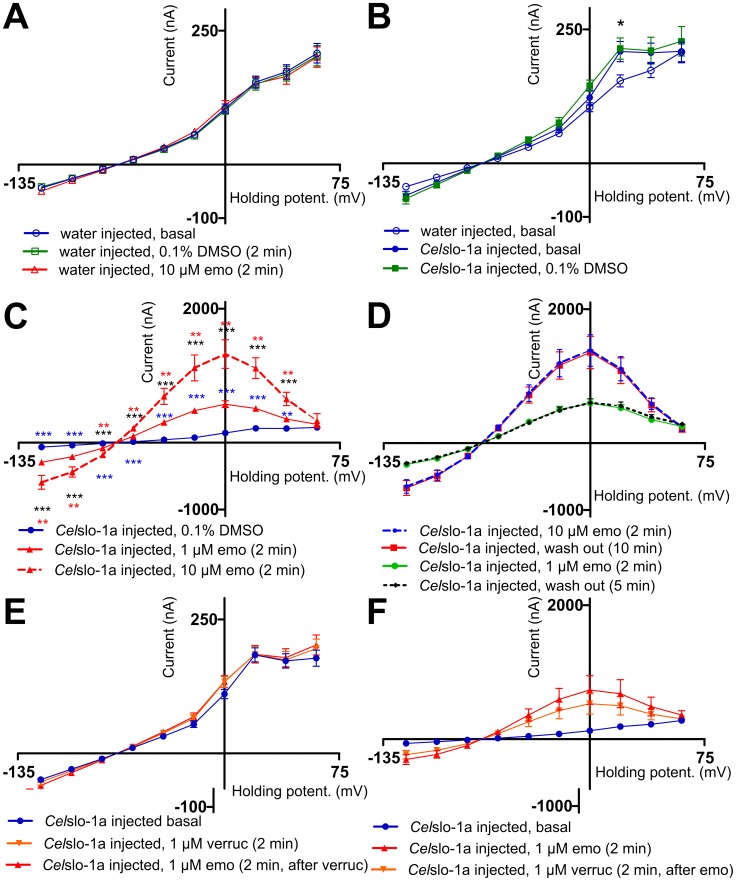
Determination of emodepside effects on current voltage curves in *Xenopus laevis* oocytes injected with *Cel*slo-1a cRNA. **A**) Current voltage curves (IVCs) (means ± SEM) were recorded in water-injected oocytes without addition of drugs (basal), after addition of the vehicle (0.1% DMSO, 0.003% Pluronic F-68) and after addition of 10 µM emodepside (emo) (n = 10). **B**) Currents obtained from oocytes injected with water (n = 10) or *Cel*slo-1a cRNA in the absence of any drug (basal, n = 8) or in the presence of vehicle (0.1% DMSO, 0.003% Pluronic F-68, n = 8). The asterisk highlights a significant difference of both curves obtained from *Cel*slo-1a cRNA injected groups of oocytes to the water-injected oocytes. **C**) Currents were recorded in *Cel*slo-1a injected oocytes at the presence of vehicle (0.1% DMSO, 0.003% Pluronic F-68), or emodepside (1 µM or 10 µM emo) (n = 10). For 10 µM emodepside the upper asterisks symbolize the comparison to 1 µM emodepside and the lower the comparison to the vehicle control. The asterisks at the 1 µM emodepside curve indicate significant differences to the control. **D**) Oocytes injected with *Cel*slo-1a were incubated with 1 µM (n = 6) or 10 µM (n = 7) emodepside (emo) before recording the IVC curves. Then, oocytes were perfused with normal frog ringer for 5 or 10 min, respectively, before a second IVC was recorded from the same oocytes (n = 7). **E**) Oocytes were preincubated in the absence of drugs (basal) or with 1 µM verruculogen (verruc) before IVCs were recorded. Then, oocytes were perfused for 2 min before 1 µM emodepside (emo) was added (n = 8). **F**) Oocytes injected with water (n = 10) or with *Cel*slo-1 cRNA (n = 6) were incubated in the absence of drugs (basal) or with 1 µM emodepside before IVCs were recorded. After perfusion with normal frog ringer for 2 min, verruculogen (verruc) was added for 2 min followed by recording of IVCs.*, p<0.05; **, p<0.01; ***, p<0.001.

Voltage-dependent currents were measured in water-injected oocytes (Fig. 4A). An inward-directed current was detected at very low negative step potentials (-120 mV to -80 mV) whereas an approximately linear increase of outward currents was detected between -60 mV and +60 mV (Fig. 4A and 5A). In water as well as in *Cel*Slo-1a or *Tmu*Slo-1.1a injected oocytes no significant differences were observed between repeatedly recorded IVCs, indicating that the oocytes were in good physiological condition. Currents detected in water-injected oocytes were not significantly influenced by the vehicle (0.1% DMSO +0.003% Pluronic F-68) or by 10 µM emodepside (Fig. 5A). In the absence of emodepside, basal IVCs between water and *Cel*Slo-1a injected oocytes were very similar. However, there was a small but significant higher current at +20 mV in *Cel*Slo-1a injected oocytes (Fig. 5B), which corresponds to the voltage of approximately 0 to +20 mV needed to open *Cel*Slo-1a at a low Ca^2+^ concentration of 10 µM [60]. This difference was also observed in the presence of the vehicle and there was no significant difference between basal IVCs and IVCs in the presence of the vehicle (Fig. 5B). Interestingly, currents did not increase further when step potentials above +20 mV were applied (Fig. 5B) and no differences to water-injected oocytes were observed at +40 and +60 mV (Fig. 5B). In addition, slightly larger negative and slightly higher positive currents were apparently observable at very low (-120 to 100 mV) and at slightly negative (-40 to -20 mV) voltages, respectively. Since the direct comparison of currents for individual voltages did not indicate significant differences, the range between -120 and -20 mV was subjected to linear regression. The runs test implemented in GraphPad Prism did not find any significant deviation from linearity for any of the data sets using the voltage range as shown in Fig. 5B. Remarkably, slopes for *Cel*Slo-1a injected oocytes were significantly higher (p<0.0001) than for water injected oocytes, no matter if DMSO was present in the medium of the *Cel*So-1a injected oocytes or not. Presence of the vehicle did not change the slope significantly (p = 0.89). The increased slopes demonstrate a voltage-independent increase in membrane conductance due to injection of *Cel*slo-1a cRNA.

No increase in currents at +20 mV was detected in oocytes injected with *Tmu*Slo-1.1a cRNA (S4 Fig.). In fact, currents measured in *Tmu*Slo-1.1a-injected oocytes were significantly lower than in water-injected oocytes at 0 mV and +20 mV step potential. At high step potentials (+40 - +60 mV), these IVCs deviated from linearity, suggesting a nonphysiological response of the oocytes. Linear regression of data between -120 and -20 mV again showed no deviation from linearity. In contrast to the data obtained after injection of *Cel*Slo-1a, the slope was not increased but even decreased by *Tmu*Slo-1a. Injection of more *Tmu*Slo-1.1a cRNA (up to concentrations of 750 ng/µL) and longer incubation between injection and electrophysiological measurements (up to six days) did not improve the results. In contrast, higher cRNA amounts only increased deviation from linearity while longer incubation times had no detectable effects. In an attempt to increase the difference for *Cel*Slo-1a or *Tmu*Slo-1.1a, oocytes were preincubated in 2 µg/ml of the Ca^2+^ ionophore A23187 (Sigma Aldrich) in Ca^2+^ free Barth's medium for 15 min followed by three washes with normal Barth's medium. However, this pretreatment did not increase the response but caused severe leak currents.

In the absence of a stimulus increasing [Ca^2+^]_i_, addition of emodepside to *Cel*Slo-1a-injected oocytes resulted in dramatically increased currents (Fig. 4C) with significantly stronger effects at 10 µM than 1 µM emodepside (Fig. 5C). Differences were significant at all step potentials except for +60 mV at both emodepside concentrations. IVCs obtained in the presence of emodepside are characterized by two very remarkable features. Firstly, currents increased even at step potentials far below the expected opening potential of Slo-1 channels, suggesting that channel opening can occur independently of membrane potential. Secondly, currents peaked at 0 mV and significantly decreased at higher step potentials, resulting in bell shaped IVCs (Fig. 5C). At the highest step potentials used (+60 mV), differences between vehicle controls and emodepside-treated oocytes were no longer significant.

Wash out of 1 µm emodepside for 5 min and wash out of 10 µM emodepside for 10 min had no effect on IVCs. Even perfusion for 25 min did not reduce the emodepside effects (n = 3). Effects of verruculogen, a specific blocker of mammalian K^+^ channels with high conductance [61], revealed that the order of addition strongly influenced the outcome. Preincubation of oocytes with verruculogen alone had no effects on IVCs; neither the slight increase in currents at +20 mV observed in *Cel*Slo-1a-injected oocytes nor the increased slope in *Cel*Slo-1 injected oocytes were blocked (compare Fig. 5B and 5E). However, addition of verruculogen before emodepside completely prevented emodepside effects on IVCs (Fig. 5E). This also shows that verruculogen effects on BK channels are not limited to mammalian channels. In contrast, when verruculogen was added to oocytes in the presence of emodepside, no decrease in emodepside-induced currents was detected (Fig. 5F).

## Discussion

The mode of action of cyclooctadepsipeptides has long been a matter of debate, with ionotropic GABA_A_ receptors [39], [62], the latrophilin receptor Lat-1 [25], [63] and the Slo-1 channel [19]-[21] consecutively deemed to be the most likely receptors. Although Slo-1 is generally considered to be the most important target based on experiments using forward and reverse genetics, there has been no direct evidence for emodepside activation of the Slo-1 channel. Responses of excitable muscle/neuronal cells to emodepside develop much slower [21], [64] than usually observed during direct gating of ionotropic receptors, which meant that indirect effects of emodepside on Slo-1 could never be completely excluded [64]. Using an oocyte expression system eliminating all nematode-related confounding factors, the present study for the first time demonstrates direct effects of emodepside on Slo-1 channels. In the presence of emodepside, highly increased currents were observed without depolarization up to a threshold of 0 mV and without any additional stimuli to artificially increase [Ca^2+^]_i_ levels. These novel findings confirm that Slo-1 is a direct target of emodepside.

The *X. laevis* expression system has been widely used to characterize the function of nematode ion channels and in particular receptors for neurotransmitters [65]-[67]. It has several advantages, including robustness, simultaneous expression of proteins (e.g., auxiliary proteins or multiple subunits) [66], and large cells which allow easy application of the two electrode voltage clamp technique (TEVC) [68]. However, there is also a drawback of using oocytes when working with Ca^2+^ responsive channels such as Slo-1. In particular, the cell is so large that it is not possible to increase the [Ca^2+^]_i_ to levels required for physiological activation of BK channels. Inside-out patch clamp recording would be required to perform this type of experiment [60]. Therefore, activation of the Slo-1 channel by simultaneously increasing the [Ca^2+^]_i_ and depolarization of the cells was not possible in this configuration. Attempts to increase [Ca^2+^]_i_ by preincubation of oocytes with the Ca^2+^ ionophore A23187 were not successful and resulted in oocytes looking very unhealthy and exhibiting large leak currents. Effects of emodepside on K^+^ currents in *A. suum* muscle flaps have been shown to be dependent extracellular Ca^2+^ [64]. Currently, data do not rule out completely that this is also the case for the experimental system used here since emodepside might itself increases [Ca^2+^]_i_ directly in *X. laevis* oocytes although it had no effect in mammalian HEK293 cells [25].

A minor activity of *Cel*Slo-1a channels was already observed in the absence of emodepside, i.e. the slope of the IVC was increased in oocytes injected with *Cel*slo-1a cRNA. This increase in membrane conductance can probably be explained by a minor number of *Cel*Slo-1a channels that are in an open state even in the absence of high [Ca^2+^]_i_ and membrane depolarization. However, strong activity of the *Cel*Slo-1a channel was only observed in the presence of emodepside, and currents mediated by *Tmu*Slo-1.1a could not be observed at all. In the absence of any other known agonist of nematode Slo-1 channels and without the ability to prove functional expression of *Tmu*Slo-1.1a by determining currents evoked by increased [Ca^2+^]_i_ and depolarization, the failure to detect emodepside effects cannot be interpreted as emodepside unresponsiveness. In fact, currents in *Tmu*Slo-1.1a-injected oocytes were lower than those observed in water-injected oocytes, suggesting that some non-physiological changes have occurred in the cells. This is supported by the fact that the IVCs in the absence of emodepside significantly deviated from linearity at high voltages, unlike currents observed in water-injected oocytes. Moreover, the slope of the IVC between -120 and -20 mV was lower than in the water-injected control oocytes. In marked contrast, *Cel*Slo-1a increased the slope and therefore membrane conductance suggesting expression of functional *Cel*Slo-1a but not *Tmu*Slo-1.1a channels. Since *T. muris* is fully susceptible to emodepside [37], [69], [70], it is unlikely that Slo-1 from this parasite should not respond to this drug. However, due to the presence of two *slo-1* paralogs in the *T. muris* genome, it cannot be ruled out that only *Tmu*Slo-1.2 is emodepside responsive. Other possibilities are that only certain T. muris Slo-1.1 splice variants respond to emodepside or that emodepside acts on *T. muris* only via the Lat-1 pathway. Optimized expression systems will be needed to distinguish between these possibilities in future experiments. For example, codon optimization might improve expression levels. Vectors that are particularly adapted for protein expression in *X. laevis* oocytes are available [71] but usage of this system resulted in extremely deteriorated oocyte morphology and oocyte rupture presumably due to toxicity of high levels of *Cel*Slo-1a. Finally, other expression systems, such as insect or mammalian cells, should be considered for *Tmu*Slo-1.1a.

In the absence of emodepside, a highly reproducible increase in current was observed at +20 mV step potential, which corresponds to the opening potential reported for *C. elegans* Slo-1a at low Ca^2+^ concentrations [60]. This small current may represent a minor depolarization-dependent activation of a few channels even in the absence of Ca^2+^ signaling. However, it remains unclear why this was not observed at more positive step potentials. Moreover, increased currents at +20 mV and increased slope of the IVC were also observed in the presence of the Slo-1 channel blocker verruculogen regardless if addition of verruculogen was followed by exposure to emodepside. Preincubation with verruculogen completely abolished the effects of emodepside. In contrast, verruculogen had no significant effect when emodepside was applied first. This suggests that both drugs bind very tightly to *Cel*Slo-1a and cannot displace each other once they have bound. High-affinity binding of emodepside to its target is also suggested by the fact that prolonged perfusion of the oocytes after removal of emodepside did not reverse channel opening. Very similar observations have been reported for effects of emodepside on *A. suum* muscle flaps [64]. Together with the slow onset of emodepside effects [64], the fact that the drug effects were not reversible might suggest that emodepside does not bind to the extracellular domain of Slo-1, but instead binds to an intracellular domain or to the transmembrane helices. The latter possibility is in agreement with its highly lipophilic nature.

Emodepside opened the *Cel*Slo-1a channel at virtually all step potentials, since currents were much higher than in the controls except at +60 mV. Unexpectedly however, the response did not increase linearly with step potential, but peaked at 0 mV, which is unusually low. The decreasing current at higher potentials and the lack of emodepside effects at +60 mV might be explained due to inhibition of Slo-1 channels by high intracellular concentrations of Ca^2+^ or Mg^2+^ as reported for mammalian BK channels (see [72] and references therein). Although [Ca^2+^]_i_ can be expected to be fairly low since there was no increased current in the absence of *Cel*Slo-1, the concentration of Mg^2+^ in *X. laevis* oocytes has been reported to be approximately 0.7 mM [73]. Whether this is also the case in the present study could not be resolved using the current voltage-clamp set up, but requires patch-clamp studies in which the concentration of these cations can be tightly controlled. Indeed, decreased currents at high step potentials in response to emodepside have not been observed for expression of *C. elegans* Slo-1 in HEK293 cells [74].

Despite their very similar morphology, genetic diversity of nematodes is known to be huge [75]. Although Slo-1 is a highly conserved channel in metazoans, it was not clear whether emodepside exerts its nematicidal effects through the same mechanisms in all nematode clades. The present study now offers the tools to compare the physiology of Slo-1 channels from different parasitic nematode lineages using optimized expression systems. The various *slo-1* splice variants identified in parasitic nematodes show that evolution has occurred at highly conserved alternative splice sites (splice region 4 with alternative exons present in nematodes, arthropods and vertebrates at the same position) along with evolution of nematode or clade-specific splice variants. Effects of these variations and of possible heteromerization of different subunits on voltage and Ca^2+^ sensitivity can now be evaluated in patch-clamp experiments.

That *B. malayi* and *B. pahangi* are rather unresponsive to emodepside *in vivo* and *in vitro* [76]–[78] cannot be easily explained by the primary structure of *Bma*Slo-1, which is virtually identical to that of other filariae. Whether this difference in susceptibility is due to target site related differences (e.g. different splice variants or combination of subunits) or due to other (not Slo-1 related) mechanisms protecting *Brugia* from emodepside effects could be answered by comparing electrophysiological properties of different splice variants between filarial species. In general, differences in emodepside responsiveness between Slo-1 channels from different species could also be used to map the emodepside binding region. Expression of hybrid channels encoded partially from a susceptible nematode and partially from a resistant species such as an arthropod followed by determination of emodepside responsiveness in electrophysiological experiments would allow to scan the sequence for those regions in the primary structure required for opening by emodepside.

The high number of splice variants encoding severely truncated Slo-1 proteins in both *Trichuris* species should be further analyzed. Truncated versions of *Cel*Slo-1 with stop codons between the S4 membrane helix and the end of the first RCK domain (regulator of conduction of potassium) have been shown to be highly resistant to emodepside [19], and a truncated version containing only the NH_2_ terminal part of a mouse Slo-1 ortholog from the start codon to the S6 transmembrane region forms a functional channel [79]. Therefore, a functional role of some of the truncated versions cannot be excluded, especially in light of the relatively high number of variants and the fact that expression levels also appear to be high in both *Trichuris* species. Truncated versions of the acetylcholine receptor subunits Unc-63 and Acr-8 have been implicated in resistance to levamisole [80] and truncated Slo-1 subunits might have dominant negative effects if they are able to heteromerize with full-length subunits but prevent the formation of a fully functional channel. That truncated channels are expressed in variants of both *Tsu*Slo.1.1 and *Tsu*Slo-1.2, and are apparently more abundant at least in some developmental stages and worm tissues than others suggests that these variants might have a physiological function, perhaps in modulating the activity of full-length gene products. The fact that temporal as well as spatial variation of splicing is *de facto* limited to *Tsu*Slo-1.1 suggests that this splice variant is under less stringent evolutionary pressure than *Tsu*Slo-1.2. A more detailed spatio-temporal picture of the expression pattern of the splice variants would help define their physiological roles.

In conclusion, these data show that the Slo-1 channel of *C. elegans* is a direct target of emodepside and that the channel is present in all important groups of parasitic nematodes of vertebrates. Sequence diversity of Slo-1 channels among these groups and within species involves several alternative splice variants and gene duplications. Interactions of subunit isoforms and effects on channel physiology and drug susceptibility are important aspects for more research on nematode neurobiology and parasitology.

## Supporting Information

S1 Fig **Summary describing electrophysiological recordings.** **A**) Individual oocytes were initially tested in the absence of drugs repeatedly to ensure that oocyte responses were stable over time (I). Effects of drugs were evaluated using the schemes (II) to (VI). **B**) Current-voltage curves (IVCs) were recorded after clamping the membrane potential to −70 mV. Then, step potentials were clamped from −120 mV to +60 mV in 20 mV steps with 3 s at −70 mV between individual voltage steps (1B). The vehicle contained 0.1% DMSO and 0.003% Pluronic F-68. Drugs and vehicle were added manually in the absence of perfusion. Emo, emodepside; VC, verruculogen; NFR, normal frog ringer.
(PDF)

S2 Fig **Comparison of Slo-1 splice variants in nematodes.** Schematic representations of different splice variants annotated in WormBase for *Caenorhabditis elegans* (CelSlo-1a-m,y,z) and *Brugia malayi* (*Bma*Slo-1c-h) in comparison with variants identified in full-length cDNAs cloned from *Dirofilaria immitis* (*Dim*Slo-1a,b), *Trichuris muris* (*Tmu*Slo-1.1a-d and *Tmu*Slo-1.2). In addition, splice variants predicted from transcriptome data for *Trichuris suis* were included. For the latter, splice variants are denominated with numbers (*e.g. Tsu*Slo-1.1.2/5/14 refers to the splice variants 2, 5 and 14 of *T. suis* Slo-1.1 which all encode the same protein but differ in alternative exons downstream of the stop codon). Related exons are shown in similar colors. *Bma*Slo-1c and *Bma*Slo-1d include a partial duplication of the alternative exon in region 4 which is marked by a black box around the partial duplicated region. Regions in which no alternative splicing was detected are indicated by red boxes. In *Trichuris* spp. Slo-1 sequences, regions corresponding to the alternatively spliced regions 5 and 6 in clade III and V nematodes are shown as an empty red box. Thin black lines indicate splice variants in which the corresponding region is missing. The location of transmembrane helices (S0-S6) and functional domains is shown at the top of the scheme. The various regions where alternative splicing was detected are enumerated (1-6). Two highly conserved phosphorylation sites for protein kinase C, located immediately before alternative splice region 4, are depicted as thin vertical lines.
(PDF)

S3 Fig **Multi-sequence-alignment of deduced proteins encoded by** ***Trichuris suis*** **slo-1.1 and slo-1.2 cDNAs in fasta format.**
(FASTA)

S4 Fig **Currents obtained from oocytes injected with water (n = 10) or** ***Tmu*** **slo-1a cRNA (n = 6) in the absence of any drug (basal) or in the presence of 10 µm emodepside (emo) (n = 6).** The asterisk highlights a significant difference between water injected oocytes and oocytes injected with *Tmu*slo-1.1a either in the absence or presence of emodepside.
(PDF)

S1 Table **Primers used for amplification of full-length slo-1 cDNA sequences, splice analysis or RACE PCRs.**
(PDF)

S2 Table **Details for Slo-1 channel protein sequences using for phylogenetic analysis and identification of splice variants.**
(PDF)
