# Supplementary figures and images for: Characterization of the Ca2+-Gated and Voltage-Dependent K+-Channel Slo-1 of Nematodes and Its Interaction with Emodepside
Source: PLoS Negl Trop Dis. 2014 Dec 18;8(12):e3401. doi: 10.1371/journal.pntd.0003401 (PMC4270693; doi:10.1371/journal.pntd.0003401)

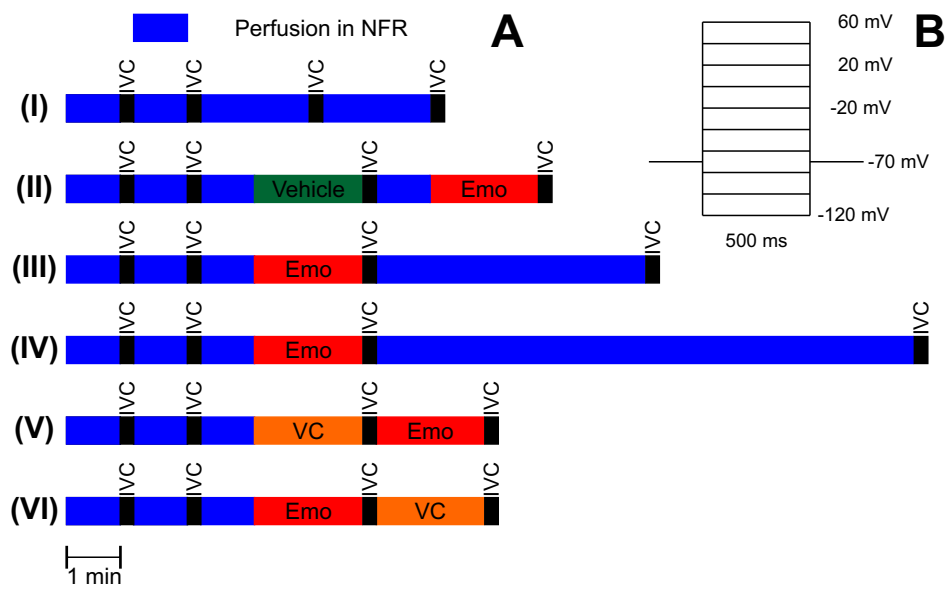

Supplement: S1 Fig — Summary describing electrophysiological recordings. A) Individual oocytes were initially tested in the absence of drugs repeatedly to ensure that oocyte responses were stable over time (I). Effects of drugs were evaluated using the schemes (II) to (VI). B) Current-voltage curves (IVCs) were recorded after clamping the membrane potential to −70 mV. Then, step potentials were clamped from −120 mV to +60 mV in 20 mV steps with 3 s at −70 mV between individual voltage steps (1B). The vehicle contained 0.1% DMSO and 0.003% Pluronic F-68. Drugs and vehicle were added manually in the absence of perfusion. Emo, emodepside; VC, verruculogen; NFR, normal frog ringer. (PDF) [file pntd.0003401.s001.pdf]

RCK domains  $\text{Ca}^{2+}$  bowl  
Voltage sensor (HMM Pfam Pf07885)

Transmembrane domains S0 S1 S2 S3 S4 S5 S6

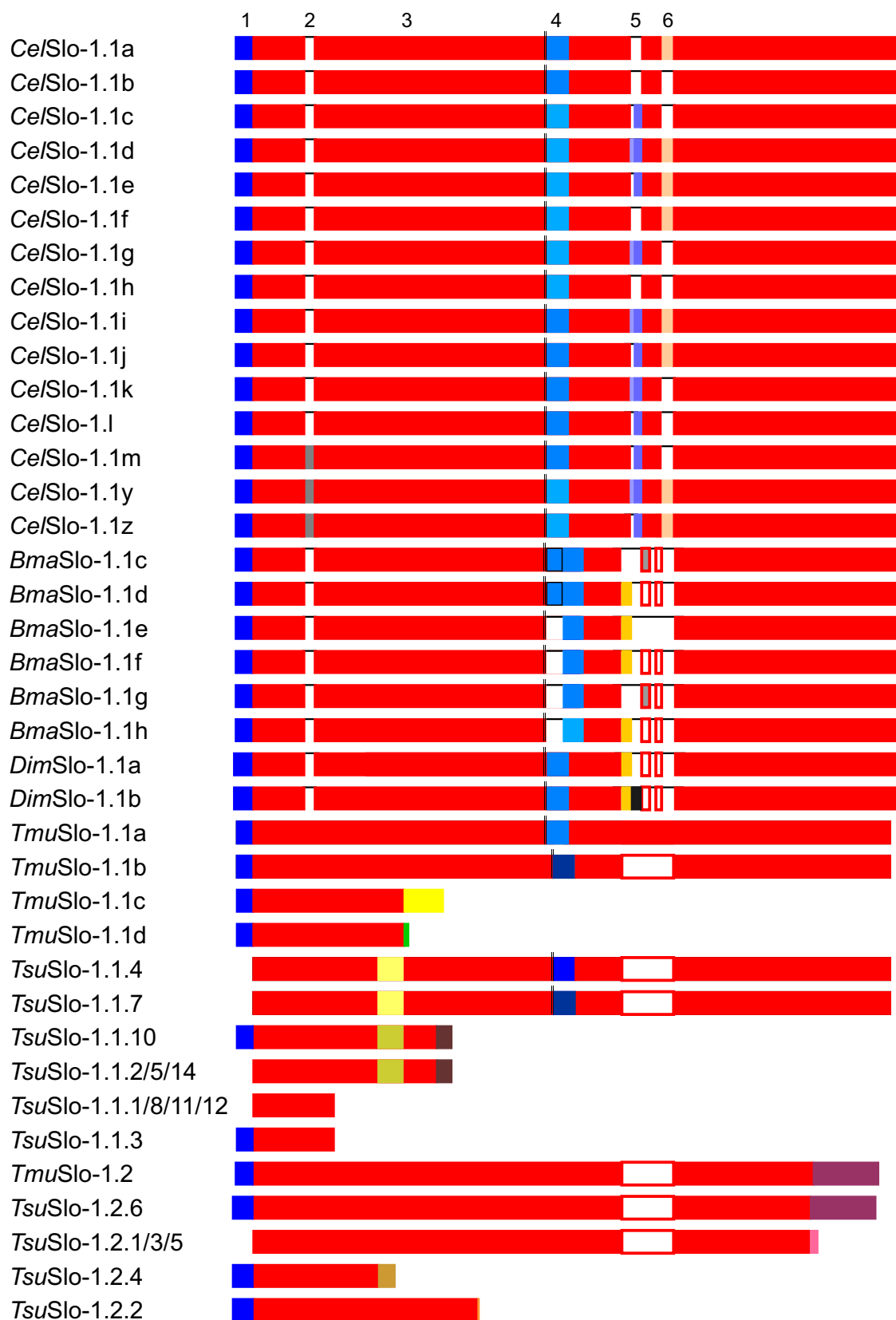

Supplement: S2 Fig — Comparison of Slo-1 splice variants in nematodes. Schematic representations of different splice variants annotated in WormBase for Caenorhabditis elegans (CelSlo-1a-m,y,z) and Brugia malayi (BmaSlo-1c-h) in comparison with variants identified in full-length cDNAs cloned from Dirofilaria immitis (DimSlo-1a,b), Trichuris muris (TmuSlo-1.1a-d and TmuSlo-1.2). In addition, splice variants predicted from transcriptome data for Trichuris suis were included. For the latter, splice variants are denominated with numbers (e.g. TsuSlo-1.1.2/5/14 refers to the splice variants 2, 5 and 14 of T. suis Slo-1.1 which all encode the same protein but differ in alternative exons downstream of the stop codon). Related exons are shown in similar colors. BmaSlo-1c and BmaSlo-1d include a partial duplication of the alternative exon in region 4 which is marked by a black box around the partial duplicated region. Regions in which no alternative splicing was detected are indicated by red boxes. In Trichuris spp. Slo-1 sequences, regions corresponding to the alternatively spliced regions 5 and 6 in clade III and V nematodes are shown as an empty red box. Thin black lines indicate splice variants in which the corresponding region is missing. The location of transmembrane helices (S0-S6) and functional domains is shown at the top of the scheme. The various regions where alternative splicing was detected are enumerated (1-6). Two highly conserved phosphorylation sites for protein kinase C, located immediately before alternative splice region 4, are depicted as thin vertical lines. (PDF) [file pntd.0003401.s002.pdf]
