# Supplementary material for: Characterization of the Ca2+-Gated and Voltage-Dependent K+-Channel Slo-1 of Nematodes and Its Interaction with Emodepside
Source: PLoS Negl Trop Dis. 2014 Dec 18;8(12):e3401. doi: 10.1371/journal.pntd.0003401 (PMC4270693; doi:10.1371/journal.pntd.0003401)
Supplement: S4 Fig — Currents obtained from oocytes injected with water (n = 10) or Tmu slo-1a cRNA (n = 6) in the absence of any drug (basal) or in the presence of 10 µm emodepside (emo) (n = 6). The asterisk highlights a significant difference between water injected oocytes and oocytes injected with Tmuslo-1.1a either in the absence or presence of emodepside. (PDF) [file pntd.0003401.s004.pdf]

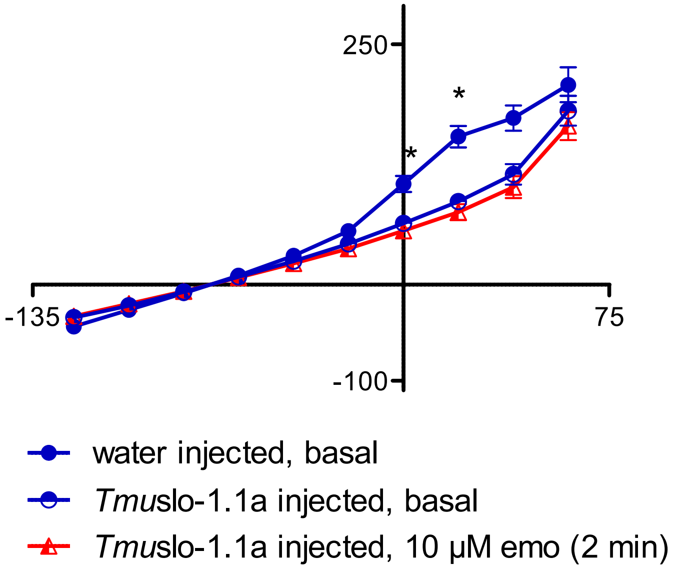

Figure S4. Currents obtained from oocytes injected with water (n=10) or *Tmu*slo-1a cRNA (n=6) in the absence of any drug (basal) or in the presence of 10 µm emodepside (emo) (n=6). The asterisk highlights a significant difference between water injected oocytes and oocytes injected with *Tmu*slo-1.1a either in the absence or presence of emodepside.
