# Supplementary material for: Characterization of the Ca2+-Gated and Voltage-Dependent K+-Channel Slo-1 of Nematodes and Its Interaction with Emodepside
Source: PLoS Negl Trop Dis. 2014 Dec 18;8(12):e3401. doi: 10.1371/journal.pntd.0003401 (PMC4270693; doi:10.1371/journal.pntd.0003401)
Supplement: S1 Table — Primers used for amplification of full-length slo-1 cDNA sequences, splice analysis or RACE PCRs. (PDF) [file pntd.0003401.s005.pdf]

Table S1. Primers used for amplification of full-length slo-1 cDNA sequences, splice analysis or RACE PCRs

Target sequence	Primer name	Primer sequence 5′ → 3′
B. malayi slo-1 full-length	Bm slo-1 Full for	GATAAATACATCGAAGGATGAGCG
	Bm slo-1 Full rev	CAGGTAAGCACAATAGGTCATAAG
D. immitis slo-1a/b full-length	Di slo-1 Full for	ATGAGCGATGTATACCATCCTGGATCCGGT
	Di slo-1 Full rev	TACAGGTAGAGCATTCTTGAGCTACATCAT
O. gutturosa slo-1 full-length	Og slo-1 Full for	ATTACCCAAGTTTGAGGTATTATTTTATTG-3
	Og slo-1 Full rev	GTAGAGCATTTTGAGCTACATCATTTTTA
A. suum slo-1 full-length	As slo-1 Full for	CATCGCCAATCCGCCGAGGAT
	As slo-1 Full rev	AGCAATTTTCATCGGGTCAT
P. equorum slo-1 full-length	Pe slo-1 Full for	CGCACCACTAGTGCCTATCA
	Pe slo-1 Full rev	AGCAATTTTCGTTGGGTCAT
T. canis slo-1 full-length	Tc slo-1 Full for	TCAGAGCTGACCGTGTCTTG
	Tc slo-1 Full rev	CGAATAGGGTAGCGGTTTCTC
T. muris slo-1.1 full-length	Tm slo-1.1 Full for	AACTTCTTCTTCCCGCGTTGGACCTTCCCCAGTT
	Tm slo-1.1 Full rev	ATCGTGGTCATGTGCTACTGTTCAATCTGTTTCGTT
T. muris slo-1.2 full-length	Tm slo-1.2 Full for	CCTTAGCCCATCGTCACAGTTCAT
	Tm slo-1.2 Full rev	TCATCGTTTCGCAGGTTCATAGGGTATACC
T. muris slo-1.1 splice analysis	Tm slo-1.1 IEA for	ACCTGTTTGCTATGCGCTCG
	Tm slo-1.1 IEA rev	CATAGGTAGTAACTGAGCCA
D. immitis slo-1 5'-RACE	Dm slo-1 5RACE1	AGGCACGAGACATATTACAATGCA
D. immitis slo-1 5'-RACE	Dm slo-1 5RACE2	TCGGATAACCGTACGGAGCTGGTGGAAATC
D. immitis slo-1 5'-RACE	Dm slo-1 5RACE3	TCGATCCACCGGATCCAGGATGGTATACAT
D. immitis slo-1 3'-RACE	Dm slo-1 3RACE1	GGTTGTATCGGTTACACGAC
D. immitis slo-1 3'-RACE	Dm slo-1 3RACE2	GTCGATTCCAATAAACGATATGTC
D. immitis slo-1 3'-RACE	Dm slo-1 3RACE3	CAAATCCACCAGCCGAACTACGAT
O. gutturosa slo-1 5'-RACE	Og slo-1 5RACE1	CAATTCATGCTTTCCTTCATCC
O. gutturosa slo-1 5'-RACE	Og slo-1 5RACE2	TATCTATTTCGTCCCGTTCTCTTTGTTGACAA
O. gutturosa slo-1 5'-RACE	Og slo-1 5RACE3	TCCAAATAACAACCAATAACATTGATACACAA
O. gutturosa slo-1 3'-RACE	Og slo-1 3RACE1	ATGGAACTGATACTGGCAGA
O. gutturosa slo-1 3'-RACE	Og slo-1 3RACE2	GCGCAGGATTACGAGGCGGA
O. gutturosa slo-1 3'-RACE	Og slo-1 3RACE3	CGAGATCGGTGCAGGATCTC
T. muris slo-1.1 5'-RACE	Tm slo-1 5RACE1	GAACTTCCTGGGTCTGGTGCTGCTGCGGTGGCGA
T. muris slo-1.1 5'-RACE	Tm slo-1.1 5RACE2	TGATAATGGAAGACAGTAGGAAGCACCAGTACTT
T. muris slo-1.1 5'-RACE	Tm slo-1.1 5RACE3	GAGGCAGCGCCTTTCTTCCTCGGTCATCTGGTCG
T. muris slo-1.1 3'-RACE	Tm slo-1.1 3RACE1	ATGCTGTCCATTGGGCTGTACAGACTGCACGACTT
T. muris slo-1.1 3'-RACE	Tm slo-1.1 3RACE2	ACTCCCAACAGACTACGTGTACGTTCTTCAACCA
T. muris slo-1.1 3'-RACE	Tm slo-1.1 3RACE3	ACGACCACGGTTTGAAGTATCCAACGAAACAGAT
T. muris slo-1.2 5'-RACE	Tm slo-1.2 5RACE1	GTTCCTGAGCACCACCGGAA
T. muris slo-1.2 5'-RACE	Tm slo-1.2 5RACE2	GTCGAGATTGCGTCGCCGATTGGTTAGC
T. muris slo-1.2 5'-RACE	Tm slo-1.2 5RACE3	GTCGAGCACGTCGTACTTGATCCGGTGA
T. muris slo-1.2 3'-RACE	Tm slo-1.2 3RACE1	CGACGACAACCTGGAATGCA
T. muris slo-1.2 3'-RACE	Tm slo-1.2 3RACE2	CAATCCAGAGTCAAGCGCCGTGATTAAG
T. muris slo-1.2 3'-RACE	Tm slo-1.2 3RACE3	ATTGCTCAATCAGCAGACGAGGTGAAGC
T. canis slo-1 5'-RACE	Tc slo-1 5RACE1	AGAAGCCTTGTGTTTGCGGCTGAATTG
T. canis slo-1 5'-RACE	Tc slo-1 5RACE2	GAATTGTGGTGATTGGTGCTGGATTT
T. canis slo-1 5'-RACE	Tc slo-1 5RACE3	TTGCACCAATAGAATGCCCTTTTCAC
T. canis slo-1 5'-RACE	Tc slo-1 5RACE4	TGTGCAATGAAGAAGCCTTGTGTTTG
T. canis slo-1 5'-RACE	Tc slo-1 5RACE5	TCCACTCCCTTCTGAGGTAATCCACA
T. canis slo-1 5'-RACE	Tc slo-1 5RACE6	ACTTCATGTCTTGCTGCTCGAACTCC
T. canis slo-1 5'-RACE	Tc slo-1 5RACE7	CGCCGAGAATGAAGAACACCATAAAA
T. canis slo-1 5'-RACE	Tc slo-1 5RACE8	CGTACTCTGCGTTGTACCACTGCTTG
T. canis slo-1 5'-RACE	Tc slo-1 5RACE9	GTTTCCACTTGAAAATTGGGGTGTGA
T. canis slo-1 5'-RACE	Tc slo-1 5RACE10	GAGAGGATGAAGACGAGCACCACAAG
T. canis slo-1 5'-RACE	Tc slo-1 5RACE11	GCGAACCCGTCTACTTCATTCTTCAA
T. canis slo-1 5'-RACE	Tc slo-1 5RACE12	GAGGTCGATTTGCTGTGAAGGACTGT
T. canis slo-1 5'-RACE	Tc slo-1 5RACE13	CAACGAAACTTGGAGGAATGGTGAAA
T. canis slo-1 5'-RACE	Tc slo-1 5RACE14	CCAAACCTTGTCCGATGCAGCTATAA
T. canis slo-1 3'-RACE	Tc slo-1 3RACE1	CGCGGCTCGGCTTACTATTACTCGCTATT

Table S1. Primers used for amplification of full-length slo-1 cDNA sequences or splice analysis (continued)

Target sequence	Primer name	Primer sequence
A. suum slo-1 5'-RACE	As slo-1 5RACE1	GAAATGGCTCACCGATTCGTACGTTA
A. suum slo-1 5'-RACE	As slo-1 5RACE2	ACTGTGCCGGTAAAGAATTCGACCTT
A. suum slo-1 5'-RACE	As slo-1 5RACE3	ACCGTACTTCTGTCTCGAACCGATCA
A. suum slo-1 5'-RACE	As slo-1 5RACE4	ATCCGCCGAGGATGAAGAATACCATA
A. suum slo-1 5'-RACE	As slo-1 5RACE5	GAGGCCGCTATAAACCGAATGAAGAA
A. suum slo-1 5'-RACE	As slo-1 5RACE6	AAAACCGAGGTCGATCTGTTGTGAAG
A. suum slo-1 5'-RACE	As slo-1 5RACE7	ACTGTCATTAATCGAAGCGCACGAAG
A. suum slo-1 5'-RACE	As slo-1 5RACE8	AGGAAGCGAAGACCTAGCCAATTACG
A. suum slo-1 5'-RACE	As slo-1 5RACE9	GCCGGTGAGGCAAACAGAAATAAAAA
A. suum slo-1 5'-RACE	As slo-1 5RACE10	TGCGATGTGTATTGGCAAAGTTCTTG
A. suum slo-1 5'-RACE	As slo-1 5RACE11	AGCGATCCAATGGAAAGGATGAAGAC
A. suum slo-1 5'-RACE	As slo-1 5RACE12	GGATGGTGAGAAGAGGTGTGTCGAGT
A. suum slo-1 3'-RACE	As slo-1 3RACE1	GATCTGCTGTTCACTCGACTCGGATT
A. suum slo-1 3'-RACE	As slo-1 3RACE2	GCTTGCGATTGAGCTAAAAGATGACG
P. equorum slo-1 5'-RACE	Pe slo-1 5RACE1	CTGGAGGCACCCTATGAAGAAAGACC
P. equorum slo-1 5'-RACE	Pe slo-1 5RACE2	AACTGTGCCGGTAAAGAACTCGACCT
P. equorum slo-1 5'-RACE	Pe slo-1 5RACE3	GAAAATCCTGAAGGAAATGGCTCACC
P. equorum slo-1 5'-RACE	Pe slo-1 5RACE4	GTCTCGAACCGATCAAGTCAGCAATC
P. equorum slo-1 5'-RACE	Pe slo-1 5RACE5	ATCCGCCGAGGATGAAGAATACCATA
P. equorum slo-1 5'-RACE	Pe slo-1 5RACE6	AATTCGGATGTGATGCGTCGTAAAAG
P. equorum slo-1 5'-RACE	Pe slo-1 5RACE7	GCGATCCAATGGAAAGGATGAAGACT
P. equorum slo-1 5'-RACE	Pe slo-1 5RACE8	AGCGATCCAATGGAAAGGATGAAGAC
P. equorum slo-1 5'-RACE	Pe slo-1 5RACE9	ATCCTCCACATCACCACAAGAAGCAT
P. equorum slo-1 5'-RACE	Pe slo-1 5RACE10	ATGGTGAGAAGAGGTGCGTCGAGTAG
P. equorum slo-1 5'-RACE	Pe slo-1 5RACE11	AGTACGTGGAATTGCATGAGGATGGT
P. equorum slo-1 3'-RACE	Pe slo-1 3RACE1	TGCTTGCGATTGAGTTAAAAGATGACG
P. equorum slo-1 3'-RACE	Pe slo-1 3RACE2	GTCGAGACACACACCAGACTGGCTAA
