# Supplementary material for: Characterization of the Ca2+-Gated and Voltage-Dependent K+-Channel Slo-1 of Nematodes and Its Interaction with Emodepside
Source: PLoS Negl Trop Dis. 2014 Dec 18;8(12):e3401. doi: 10.1371/journal.pntd.0003401 (PMC4270693; doi:10.1371/journal.pntd.0003401)
Supplement: S2 Table — Details for Slo-1 channel protein sequences using for phylogenetic analysis and identification of splice variants. (PDF) [file pntd.0003401.s006.pdf]

Table S2. Details for Slo-1 channel protein sequences using for phylogenetic analysis and identification of splice variants

Species	Systematic position	Protein	Amino acid length	Molecular Weight (kDa)	pI	Accession no.[a]
Caenorhabditis elegans	Chromadorea Rhabditida Rhabditoidea clade V	*Cel*Slo-1a	1140	129.5	5.75	NP_001024259
Caenorhabditis elegans	Chromadorea Rhabditida Rhabditoidea clade V	*Cel*Slo-1b	1118	127.0	5.67	NP_001024260
Caenorhabditis elegans	Chromadorea Rhabditida Rhabditoidea clade V	*Cel*Slo-1c	1131	128.6	5.58	NP_001024261
Caenorhabditis briggsae	Chromadorea Rhabditida Rhabditoidea clade V	*Cbr*Slo-1	1142	129.7	5.69	XP_2638493
Caenorhabditis remanei	Chromadorea Rhabditida Rhabditoidea clade V	*Cre*Slo-1	1167	132.6	5.77	XP_003094347
Haemonchus contortus	Chromadorea Rhabditida Rhabditoidea clade V	*Hco*Slo-1	1105	125.2	5.77	ABS45068
Cooperia oncophora	Chromadorea Rhabditida Rhabditoidea clade V	*Con*Slo-1	1111	125.9	5.80	ABS45069

Species	Systematic position	Protein	Amino acid length	Molecular Weight (kDa)	pI	Accession no.[a]
Ancylostoma caninum	Chromadorea<br>Rhabditida<br>Rhabditoidea<br>clade V	*Aca*Slo-1	1116	126.7	5.44	ACC68842
Pristionchus pacificus	Chromadorea<br>Rhabditida<br>Rhabditoidea<br>clade V	*Ppa*Slo-1	1188	134.1	5.56	PPA29145[b]
Strongyloides ratti	Chromodorea<br>Rhabditida<br>Panagrolaimoidea<br>Clade IV	*Sra*Slo-1	1170	131.4	5.51	Sr321_0X0013600.t1[c]
Meloidogyne incognita	Chromodorea<br>Tylenchida<br>Tylenchoidea<br>CladeIV	*MinSLO-1*	1120	126.4	5.39	Minc04076a[d]
Dirofilaria immitis	Chromadorea<br>Spirurida<br>clade III	*Dim*Slo-1a	1119	126.7	5.27	AFH88396
Dirofilaria immitis	Chromadorea<br>Spirurida<br>clade III	*Dim*Slo-1b	1104	125.0	5.44	AFX93730
Onchocerca gutturosa	Chromadorea<br>Spirurida<br>clade III	*Ogu*Slo-1	1119	126.8	5.27	ADY18306
Brugia malayi	Chromadorea<br>Spirurida<br>clade III	*Bma*Slo-1c	1115	126.3	5.19	BM6719c[b]
Brugia malayi	Chromadorea<br>Spirurida<br>clade III	*Bma*Slo-1d	1132	128.2	5.39	BM6719d[b]
Brugia malayi	Chromadorea<br>Spirurida<br>clade III	*Bma*Slo-1e	1079	122.3	5.30	BM6719e[b]

Species	Systematic position	Protein	Amino acid length	Molecular Weight (kDa)	pI	Accession no.[a]
Brugia malayi	Chromadorea<br>Spirurida<br>clade III	*Bma*Slo-1f	1104	125.1	5.40	BM6719f[b]<br>KJ531222
Brugia malayi	Chromadorea<br>Spirurida<br>clade III	*Bma*Slo-1g	1087	123.2	5.19	BM6719g[b]
Brugia malayi	Chromadorea<br>Spirurida<br>clade III	*Bma*Slo-1h	1104	125.0	5.44	BM6719h[b]
Ascaris suum	Chromadorea<br>Ascaridoidea<br>clade III	*Asu*Slo-1	1117	126.4	5.04	ACC68842
Parascaris equorum	Chromadorea<br>Ascaridoidea<br>clade III	*Peq*Slo-1	1108	125.2	5.51	ACC68843
Toxocara canis	Chromadorea<br>Ascaridoidea<br>clade III	*Tca*Slo-1	1123	126.9	5.38	ACJ64718
Trichuris muris	Enoplea<br>Trichocephalida<br>clade I	*Tmu*Slo-1.1a	1151	130.1	5.70	AEB96250
Trichuris muris	Enoplea<br>Trichocephalida<br>clade I	*Tmu*Slo-1.1b	1151	130.2	5.67	KJ531218
Trichuris muris	Enoplea<br>Trichocephalida<br>clade I	*Tmu*Slo-1.1c	346	41.5	6.21	KJ531219
Trichuris muris	Enoplea<br>Trichocephalida<br>clade I	*Tmu*Slo-1.1d	303	34.7	6.48	KJ531220

Species	Systematic position	Protein	Amino acid length	Molecular Weight (kDa)	pI	Accession no.[a]
Trichuris muris	Enoplea<br>Trichocephalida<br>clade I	*Tmu*Slo-1.2	1123	126.9	5.59	KJ531221
Trichinella spiralis	Enoplea<br>Trichocephalida<br>clade I	*Tsp*Slo-1.1	1134	128.5	5.57	XP_003370273<br>XP_003370274[e]
Trichinella spiralis	Enoplea<br>Trichocephalida<br>clade I	*Tsp*Slo-1.2	1129	128.0	5.2	XP_003370270<br>XP_003370271<br>XP_003370272[e]
Daphnia pulex	Ecdysozoa<br>Crustacea<br>Branchiapoda	*Dpu*Slo-1	1028	115.7	5.60	EFX85873
Pediculus humanus corporis	Ecdysozoa<br>Insecta<br>Anoplura	*Phu*Slo-1	1141	127.9	5.28	XP_002425826
Anopheles gambiae	Ecdysozoa<br>Insecta<br>Diptera	*Aga*Slo-1	1154	129.1	5.19	XP_313505.5
Drosophila melanogaster	Ecdysozoa<br>Insecta<br>Diptera	*Dme*Slo-1Q	1175	130.3	5.13	NP_001014651
Drosophila melanogaster	Ecdysozoa<br>Arthropoda<br>Diptera	*Dme*Slo-1S	1210	134.4	5.48	NP_001163712
Aplysia californica	Lophotrochozoa<br>Mollusca<br>Gastropoda	*Acal*Slo-1	1070	120.2	6.34	AAR27959
Gallus gallus	Vertebrata<br>Aves	*Gga*Slo-1	1140	128.1	6.60	AAD16633

Species	Systematic position	Protein	Amino acid length	Molecular Weight (kDa)	pI	Accession no.[a]
Bos taurus	Vertebrata Mammalia Ruminantia	*Bta*Slo-1	1166	130.1	6.31	NP_777105
Homo sapiens	Vertebrata Mammalia Primates	*Hsa*Slo-1	1236	135.6	6.64	NP_001154824

[a]If not further specified protein accession numbers from GenBank are given.

[b]In WormBase.

[c]In Sanger *S. ratti* genome database.

[d]In INRA *M. incognita* genome database.

[e]A full-length consensus sequence was built from these entries.
